# Effects of the corona measures on the life of children and adolescents: Results of the German HBSC study 2022

**DOI:** 10.25646/13002

**Published:** 2025-03-05

**Authors:** Franziska Reiß, Veronika Ottová-Jordan, Ludwig Bilz, Irene Moor, Kevin Dadaczynski, Ronja Maren Helmchen, Theresa Schierl, Saskia Sendatzki, Katharina Rathmann, Anne Kaman, HBSC Study Group Germany, Ulrike Ravens-Sieberer

**Affiliations:** 1 University Medical Center Hamburg-Eppendorf, Center for Psychosocial Medicine, Department of Child and Adolescent Psychiatry, Psychotherapy and Psychosomatics, Research Section Child Public Health, Hamburg, Germany; 2 Brandenburg University of Technology Cottbus-Senftenberg, Institute of Health, Senftenberg, Germany; 3 Martin Luther University Halle-Wittenberg, Medical Faculty, Profile Center for Health Sciences (PZG), Institute of Medical Sociology, Halle (Saale), Germany; 4 Fulda University of Applied Sciences, Department of Health Sciences, Fulda, Germany; 5 Leuphana University Lüneburg, Center for Applied Health Sciences, Lüneburg, Germany; 6 Fulda University of Applied Sciences, Public Health Centre Fulda, Fulda, Germany; 7 Technical University Munich, School of Medicine and Health, Chair of Social Determinants of Health, Munich, Germany

**Keywords:** HBSC, COVID-19 pandemic, Children, Adolescents, (Mental) health, Social relationships, Germany

## Abstract

**Background:**

The containment measures of the COVID-19 pandemic have changed many people’s daily lives. The study examines how children and adolescents assess the impact of the coronavirus measures on various areas of life.

**Methods:**

Overall, *N* = 6,475 students (11 – 15 years) in Germany took part in the representative ‘Health Behavior in School-aged Children (HBSC)’ study in 2022. Logistic regressions were performed to investigate the relationships between the perceived impact of the coronavirus measures on various areas of their lives by age, gender and family wealth.

**Results:**

Two years after the onset of the pandemic, children and adolescents reported both, positive and negative effects of the coronavirus measures. About a half of those surveyed perceived positive effects regarding social relationships. In contrast, approximately one in three respondents reported a deterioration in mental health and school performance. In particular, 11-year-olds, boys and wealthy students reported more often positive effects.

**Conclusions:**

The positive effects of the pandemic on the lives of children and adolescents in individual areas and the resilience (resistance) that is emerging here, as well as the results on more negative assessments of adolescents, girls and respondents with lower family wealth, can be used as a starting point for a needs-oriented and target group-specific health promotion in times of crisis. Future research should focus on the pandemic’s long-term effects on young people’s development.

## 1. Introduction

In spring 2020, when the COVID-19 pandemic began, children and adolescents faced enormous challenges that demanded high adaptability in many areas of life. Not only the fear of infection and concerns about the possible health consequences of an infection with the coronavirus (SARS-CoV-2), but also the containment measures put the adult population and children and adolescents in particular in a (collective) state of emergency. The latter included school closures [1], as well as social contact restrictions and closed leisure and sports facilities.

Childhood and adolescence represent a very sensitive (developmental) phase in which important psychosocial developmental tasks are mastered [2, 3]. The pandemic massively disrupted these genuine developmental tasks. The subsequent consequences of the pandemic (school closures, homeschooling) as well as other global crises (such as the Ukraine War and climate change) saw children and adolescents facing new challenges and burdens [4]. The ongoing ‘crisis mode’ required adults and adolescents to quickly adapt to the new demands, coping tasks and circumstances.

Previous studies have shown that school closures were a significant stressor at the beginning of the pandemic, both for children and young people [5] and their parents [6]. In the COrona and PSYche (COPSY) study [7], more than 70 % of children and young people stated that they felt stressed by the pandemic and the associated changes. The lack of contact with friends due to isolation and the lack of sports or physical activity were also negatively associated with children’s and adolescents’ mental health, particularly in connection with depression and anxiety disorders [8]. Some studies indicate that the mental health of children and adolescents in Germany have deteriorated during the pandemic [9] and that recovery effects occur only slowly [10]. Psychosomatic complaints, such as anxiety, sleeping difficulties, abdominal pain and headaches have increased significantly in recent years [11], and students from all types of schools have reported a higher level of stress due to their schoolwork. Systematic reviews have shown that in terms of mental health, especially girls and older adolescents were more frequently affected by depression and anxiety [12, 13].

Gender and age-related differences were also observed in health behaviour [3]. Boys were more physically active than girls, but gender differences decreased with increasing age [9]. Furthermore, socioeconomically disadvantaged students were more affected by the impact of the pandemic [3], had greater learning deficits [14] and showed poorer (mental) health [15] and worse health behaviours [16].

Apart from the adverse effects, there are a few studies which report positive developments. In a longitudinal study from Lithuania [17], various development profiles for 12- to 16-year-old adolescents were mapped over the course of two measurement periods (2019 and 2020). In one group (9.7 %), improvements in prosocial behaviour could be observed. However, group-related differences were not examined, including sociodemographic and socioeconomic variables. In a Canadian cross-sectional study of 14- to 25-year-olds [18], researchers not only found negative effects, particularly in terms of mental health, but also a high probability of high subjective well-being for 36.6 % (‘resilient’). Respondents in the ‘resilient’ group were younger, male and showed a higher level of resilience. Further evidence for differently directed effects of the COVID-19 pandemic can be found in a Chinese study of 12- to 24-year-olds [19], in which latent profile analyses were used to form three groups (limited, partial and intense positive changes). Also here, associations between young age and higher resilience could be confirmed with group membership of those with intense positive changes.

**Key messages**

► Children’s and adolescents’ viewpoint on coronavirus measures was rarely examined. Results reveal both, negative and positive consequences on the lives of young people.
► Positive effects of the coronavirus measures were most frequently reported for social relationships with family and friends.
► Negative effects of the coronavirus measures were most frequently seen in mental health and school performance.
► Girls and older students were more likely to report adverse effects of the coronavirus measures on most areas of life.
► Wealthier students were more likely to report positive effects of the coronavirus measures in most areas of life.

The mentioned studies deal primarily with the consequences of the COVID-19 pandemic for society and the lives of adolescents, see e.g. [1, 14, 20]. Less is known about how children and adolescents look (back) on the pandemic and how they assess the impact of the pandemic on their own life situation. The available findings primarily come from qualitative studies that approach the assessment of the coronavirus pandemic from the narrative perspective of adolescents and show what consequences social isolation, loss of autonomy, and changes in the usual daily structure had for adolescents [21–24].

When looking back on a crisis, taking a subjective view is essential. This article focuses on the perspective of children and adolescents and shows how they assess the impact of the coronavirus measures on various areas of their lives two years after the outbreak of the pandemic. Based on the state of research presented, age, gender and family wealth are considered.

From a public health perspective, the topic is relevant as it addresses all levels of the model of social determinants of health, such as the central socialisation contexts and living environments of children and adolescents (i.e. family, school, leisure time, friends) [25], but also socioeconomic conditions (financial situation) and values (future expectations) that influenced their lives during the pandemic. Thus, the present database offers the opportunity to map the various dimensions and areas of life of children and adolescents and examine them using a comprehensive school-based database.

The research questions to be answered in this article are:

1. How do students assess the impact of the coronavirus measures on different aspects of their lives two years after the outbreak of the pandemic?
2. To what extent do age, gender and socioeconomic differences exist in assessing the impact of the pandemic?

## 2. Methods

### 2.1 Sample design and study implementation

The ‘Health Behavior in School-aged Children’ (HBSC) study is a cross-sectional study that surveys students at approximately 11, 13 and 15 years of age (mean deviation of 0.5 years) in schools every four years. Meanwhile, the study is conducted in 51 countries under the auspices of the World Health Organization (WHO) [26]. In Germany, the age groups mainly comprise grades 5, 7 and 9. A total of 174 schools from all 16 federal states took part in the German HBSC Study 2022, whereby a stratified random sample was drawn to ensure a representative distribution in terms of geographical location. The response rate for the survey was 8.4 % for the schools contacted and 56.8 % for the participating students [27]. The invited schools were drawn as a cluster sample from the population of all mainstream schools in Germany. In order to obtain a representative estimate (close to the distribution of the population), the school size and the percentage distribution of students stratified by school type were included in the sampling (Probability Proportional to Size (PPS) design).

The HBSC study was carried out using a paper or online questionnaire, which the students completed themselves. The study was approved by the responsible ministries or state education authorities in all federal states (except North Rhine-Westphalia, as schools there decide autonomously whether to participate). Participation was voluntary in the school context, provided written consent was obtained from students and their parents/guardians on the survey day. Further information on the design, methodology and results of the German HBSC Study can be found elsewhere [27].

### 2.2 Survey methods

The international HBSC consortium developed the COVID-19 Impact Scale to assess the impact of the pandemic on different areas of students’ lives. The question is: ‘Since the beginning of the Corona pandemic, many people’s lives have been affected (i.e. lockdowns, school closures, distance learning, contact restrictions, social distancing and hygiene rules, closures of leisure and sports facilities, travel restrictions). What impact have these measures had on the following aspects of your life? (A negative impact means that they have made things worse, a positive impact means that they have made things better)’. The scale comprises ten items on the impact of the COVID-19 measures on: 1) life in general, 2) overall health, 3) relationships with family, 4) relationships with friends, 5) mental health, 6) school performance, 7) physical activity, 8) eating behaviour, 9) future expectations and 10) family finances. The answers were given on a five-point Likert scale, ranging from ‘very negative’ (1) to ‘very positive’ (5), with a neutral value (i.e. (3) ‘neither positive nor negative’) as the middle category [28]. For the descriptive and bivariate analyses, the response options were combined as follows: ‘very negative’ and ‘fairly negative’ merged into ‘negative impact’, ‘neither positive nor negative’ remained as the middle category and ‘fairly positive’ and ‘very positive’ were combined as ‘positive impact’.

**Infobox**

**HBSC 2022**

**Data holder:** HBSC Study Group Germany

**Objective:** The study aims to analyse the health and health behaviour of students. Continuous health monitoring through the HBSC study contributes to informing decision-makers in policy and practice about the current fields in prevention and health promotion in childhood and adolescence. A particular focus is on the influencing factors and the social contexts of health in the young generation.

**Study design:** Cross-sectional survey by written questionnaire every four years

**Population:** Students with average ages 11, 13, and 15 years

**Sampling:** Observation units are schools and the class groups are clustered within them. A cluster sample was drawn from the population of all state general education schools in Germany. In order to obtain a representative estimate (close to the distribution of the population), school size and the percentage distribution of students were included in the sampling, stratified by school type and federal state (Probability Proportional to Size (PPS) design).

**Data collection period:** March – November 2022

**Sample size:** 6,475 students

More information in German can be found at https://hbsc-germany.de/

Sociodemographic characteristics included age, gender and socioeconomic status. Age was collected categorically for 11-, 13- and 15-year-olds. Information on gender could be provided as ‘girl’, ‘boy’, and ‘diverse’.

Socioeconomic status was measured using the Family Affluence Scale III, which consists of six items and was developed and validated as part of the HBSC study [29]. The scale is suitable for participants aged 11 to 15 years and measures various aspects of family wealth (car ownership, own (bed)room, vacations with the family, computer ownership, number of bathrooms, owning a dishwasher). A summative index was formed from these six items, which was converted using a RIDIT (Relative to an Identified Distribution Integral Transformation) calculation and then divided into three groups low (lower approx. 20 %), medium (middle approx. 60 %) and high (top approx. 20 %) family wealth using a quintile classification. Further information can be found in Moor et al. [16].

### 2.3 Statistical methods

In the first step, the students’ answers to the ten questions of the COVID-19 Impact Scale were analysed descriptively, once across the entire sample and then separately for the subgroups according to age, gender and family wealth. Cramer’s V was used to determine the size of the differences and interpreted according to the following conventions: small (V = 0.1), medium (V = 0.3) and strong (V = 0.5) [30]. In order to be able to interpret the results, an effect size of at least small was required.

Logistic regression analyses were calculated to validate these relationships multivariately. The answers to the ten questions were used as criterion variables. For subjectively perceived positive effects, the response categories ‘very positive’ and ‘fairly positive’ (1) were compared with all other response categories (0); for perceived adverse effects, the response categories ‘very negative’ and ‘fairly negative’ (1) were compared with all other categories (0). The age group (reference category: 11 years), gender (reference category: boys, excluding children and adolescents with gender diversity) and family wealth (reference category: top quintile) served as categorical predictor variables in the overall sample. Due to the low cell populations compared to the girls and boys, children and adolescents who classified themselves as ‘diverse’ had to be excluded from these analyses with the overall sample.

To make reliable statements for the group of children and adolescents with gender diversity, additional logistic regression analyses were carried out in a gender-specific sub-sample, taking into account the triple-graded gender factor. For this purpose, 300 girls and 300 boys were randomly drawn from the overall sample and matched with the children and adolescents with gender diversity. The aim of this procedure was to analyse a sample with balanced cell populations in terms of gender.

Odds ratios (OR), significance levels and the 95 % confidence interval are reported. All analyses were performed with IBM SPSS Statistics 29.

## 3. Results

An overview of the sample characteristics differentiated by gender, age and socioeconomic status is shown in Table 1. The sample comprises a total of *N* = 6,475 children and adolescents with a balanced age and gender distribution and a mean age of 13.46 years (standard deviation (*SD*) = 1.68 years). The youngest person was 10.83 and the oldest person was 16.33 years old.

###  

#### Assessment of the COVID-19 measures on various areas of life

**Figure 1:**
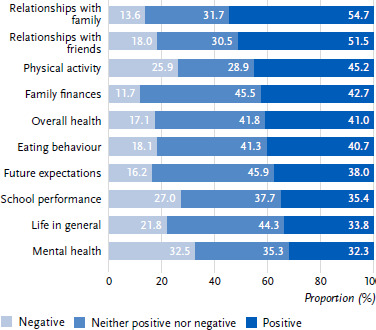
Perceived impact of the coronavirus measures on various areas of life (in %, *N* = 5,692 – 5,813)*. Source: HBSC Germany 2022 *Rounding deviations possible

The effects of the coronavirus measures were assessed by children and young people in individual areas of life as either positive, negative or neutral. The majority of adolescents rated the effects on social relationships, i.e. with family (54.7 %) and friends (51.5 %), but also on physical activity (45.2 %), such as sports, cycling and walking, as relatively positive (Figure 1).

The effects of the coronavirus measures on future expectations (45.9 %), e.g. about exams and jobs, family finances (45.5 %) and life in general (44.3 %), were most frequently assessed as neutral, i.e. neither positive nor negative.

Adverse effects of the coronavirus measures were reported much less frequently, most often for mental health (32.5 %), e.g. in relation to stress and dealing with one’s feelings. The effects on school performance (27.0 %) and physical activity (25.9 %) were also described as negative by around a quarter of the respondents in each case.

#### Age-specific differences in the assessment of the coronavirus measures

**Figure 2:**
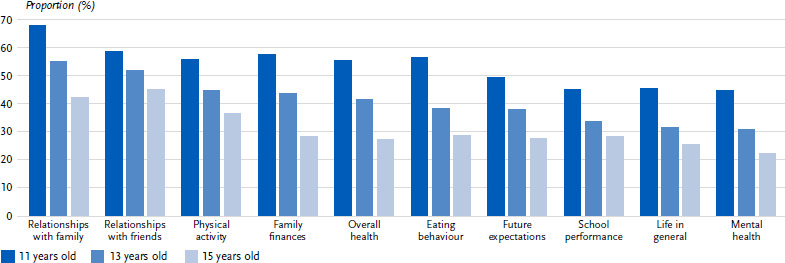
Positively perceived effects of the coronavirus measures on various areas of life by age group (figures in %, *n* = 1,806–1,860 11-year-olds, *n* = 1,952–1,996 13-year-olds, *n* = 1,934–1,952 15-year-olds)*. Source: HBSC Germany 2022 *All differences shown are statistically significant (p < 0.001).

In terms of age, younger students were significantly more likely to give a positive assessment of the effects of the coronavirus measures in all areas of their life than older students (Figure 2, Annex Table 1). The most apparent age-specific differences were seen in the assessment of social relationships with family (11 years: 67.6 % vs. 15 years: 42.0 %; *p* < 0.001; V = 0.158) and with friends (11 years: 58.3 % vs. 15 years: 45.0 %; *p* < 0.001; V = 0.098), the family’s financial situation (11 years: 57.1 % vs. 15 years: 28.4 %; *p* < 0.001; V = 0.176), eating behaviour (11 years: 56.2 % vs. 15 years: 28.6 %; *p* < 0.001; V = 0.165) and overall health (11 years: 55.2 % vs. 15 years: 27.3 %; *p* < 0.001; V = 0.164). However, the effect sizes were small for all areas of life. No clear pattern could be derived for the negative effects.

#### Gender-specific differences in the assessment of the coronavirus measures

**Figure 3:**
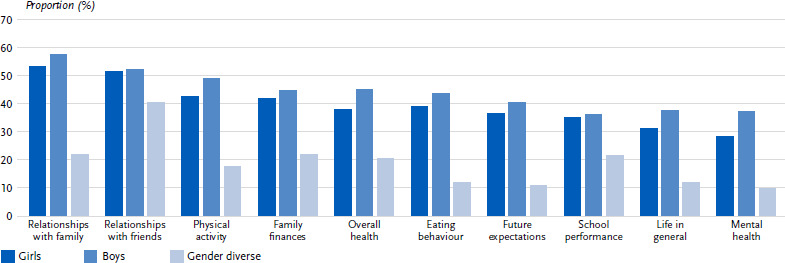
Positively perceived effects of the coronavirus measures on various areas of life by gender (figures in %, *n* = 2,841 – 2,901 girls, *n* = 2,753 – 2,816 boys, *n* = 101 –102 gender-diverse)^*^. Source: HBSC Germany 2022 ^*^Except the category ‘Relationship with friends’, all the differences shown are statistically significant (*p* < 0.001).

A gender comparison showed that boys were slightly more likely to rate the effects of the coronavirus measures positively compared to girls or gender-diverse children and adolescents (Figure 3, Annex Table 1). However, the effect sizes were so small that interpretable gender-specific differences only emerged in the area of mental health. Here, boys were significantly more likely to rate the effects of the coronavirus measures on mental health as positive compared to girls (37.0 % vs. 28.4 %) or gender-diverse children and adolescents (9.9%) (*p* < 0.001; V = 0.089).

#### Differences in the assessment of the coronavirus measures according to family affluence

**Figure 4:**
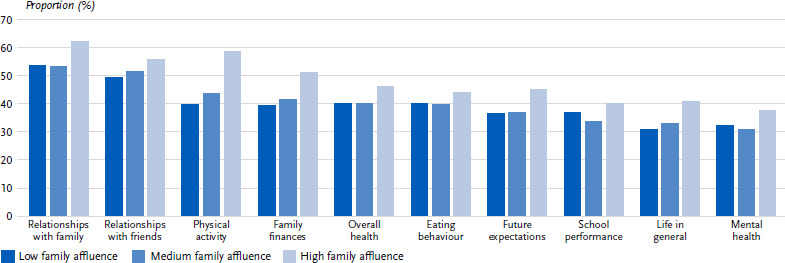
Positively perceived effects of the coronavirus measures on various areas of life according to family affluence (figures in %, *n* = 829 – 849 high family affluence, *n* = 3,752 – 3,823 medium family affluence, *n* = 988 –1,013 low family affluence). Source: HBSC Germany 2022

The differentiation by family affluence in the descriptive analysis showed that the effects of the coronavirus measures were significantly more frequently described as negative in some areas by children and adolescents with low family affluence (Figure 4, Annex Table 1). However, the effect sizes were low in all areas of life. The only trend that emerged was that of children and adolescents with a high or medium level of family affluence perceiving the effects of the coronavirus measures on physical activity more often as positive than those with a low level of family affluence (high level of family affluence: 58.5 %, medium level of family affluence: 43.5 %, low level of family affluence: 39.7 %; *p* < 0.001; V = 0.086).

#### Multivariate results

For multivariate validation of the relationships between gender, age and family affluence and the effects of the corona measures, binary-logistic regressions were performed to investigate the risk of experiencing negative and the chance of experiencing positive effects of the corona measures.

The results made it clear that the risk of experiencing negative effects of the coronavirus measures on various areas of life differs significantly according to age, gender and family affluence (Annex Table 2). For most areas of life, there were differences to the detriment of 15-year-olds compared to 11-year-olds, girls compared to boys and respondents from less affluent families compared to children and adolescents from more affluent families. Compared to 11-year-olds, 15-year-olds showed a significantly higher risk of a negative assessment of the effects of the coronavirus measures on mental health (OR = 2.68), school performance (OR = 2.42) and eating behaviour (OR = 1.96). Compared to boys, girls showed a significantly higher risk of a negative assessment of the effects of the coronavirus measures on mental health (OR = 2.43) and eating behaviour (OR = 1.73). Respondents with low family affluence showed a significantly increased risk of a negative assessment of the effects of the coronavirus measures on physical activity (OR = 2.11), the family’s financial situation (OR = 2.07) and relationships with the family (OR = 1.89) compared to those with high family affluence.

The regression results for predicting the experience of positive influences of the coronavirus measures on various areas of life showed that the chances of a positive assessment are also unevenly distributed according to age and gender (Annex Table 3). In the multivariate results for 15-year-olds compared to 11-year-olds, a significantly lower chance of a positive experience of the effects of the pandemic on, for example, mental health (OR = 0.35), the financial situation (OR = 0.29), eating behaviour (OR = 0.31) and physical activity (OR = 0.46) was observed. In the overall sample, girls also showed a lower chance of experiencing the effects of the pandemic positively compared to boys, including in the area of mental health (girls: OR = 0.68). With regard to the distribution of opportunities, it also became clear that respondents with low (OR = 0.61) and medium family affluence (OR = 0.67) had a lower chance of experiencing the effects of the pandemic positively compared to wealthier children and adolescents, for example in terms of the financial situation.

The analyses with the gender-specific subsample, including children and adolescents with gender diversity, showed that this group consistently reported more negative and fewer positive influences of the pandemic on the ten areas of life surveyed compared to girls and especially boys (Annex Table 4 and Annex Table 5). Regarding the negative influences, the odds ratios for this group were higher than those for each, girls and boys, they differed statistically significantly from the reference group (boys) with two exceptions (relationships with friends, financial situation of the family) and were particularly high for mental health (OR = 5.26) and overall health (OR = 4.55). The odds ratios for the children and adolescents with gender diversity were also always the lowest for the positive effects, differed statistically significantly from the reference group (boys) with two exceptions (relationships with friends, school performance) and were generally low. Particularly strong associations were found for mental health (OR = 0.3) and eating behaviour (OR = 0.31).

## 4. Discussion

Two years after the outbreak of the COVID-19 pandemic, the children and adolescents surveyed in the HBSC study reported that the coronavirus measures had both positive and negative effects on various areas of their lives. The differently directed effects known from international studies [17–19] could thus be confirmed also for children and adolescents in Germany. In this study, the effects were more frequently assessed as neutral or positive, although there were clear differences in the assessment of individual areas of life according to age, gender and family affluence. Younger children aged 11 years, boys and affluent children and adolescents in particular reported that the pandemic also had a positive impact on their lives. Particularly negative effects of the coronavirus measures were reported by children and adolescents with gender diversity, who were taken into acoount separately for the first time as part of the study.

In general, around half of the children and adolescents stated that their social relationships with friends and family had improved. About 45 % neither saw an improvement nor a deterioration in their financial situation or life as a whole, whereas around one in three respondents reported a deterioration in both, their mental health and school performance, as a result of the coronavirus measures.

The neutral to positive assessment of the pandemic in this study is consistent with the results of a study by the German Youth Institute [31], in which the majority of parents of 3- to 15-year-olds reported during the coronavirus pandemic that their children were coping relatively well to very well with the coronavirus crisis. Positive experience reports, especially in the first lockdown (spring 2020), are probably due to the fact that parents had more time for joint activities [6] and spent more time with their children [32]. This reflects the importance of good social contacts for coping with crisis situations [22, 23]. A stable relationship with an adult is an important protective factor for children and adolescents in difficult life situations [33] and good family cohesion has a protective effect, which could mitigate the stress caused by the pandemic [34, 35]. However, this protective factor was primarily evident in families with a higher socioeconomic status [36]. Another explanation for the positive perceptions of the effects of the pandemic reported here could be due to the timing of the survey, which was approximately two years after the onset of the pandemic. At this point, the pandemic had already passed the peak of the coronavirus wave and the strict coronavirus measures had been relaxed. This loosening of the restrictions permitted more ‘old normality’. Thus, the retrospective assessment can be explained by memory psychological effects on the one hand and as an indication of healthy emotional processing of challenging experiences on the other. These can go hand in hand with selective attention [37] as well as a positivisation of memories in order to improve self-assessment and well-being [38]. The so-called ‘fading affect bias’, according to which emotions associated with unpleasant past events fade more quickly in memory than emotions associated with pleasant past events, certainly also plays a role here [39].

In contrast, the children and adolescents in this study reported negative consequences of the pandemic in the areas of mental health and school performance. Previous studies confirm a deterioration in the mental health of young people during the pandemic [23] and major stress caused by the limited care and schooling [35]. In addition to socially unevenly distributed technical challenges resulting from the change from face-to-face to distance learning, the lack of space for learning, the lack of interaction with peers and school staff and the resulting loss of engagement and motivation to learn are possible causes [36, 40].

The age differences clearly observed in this study were also evident in other studies, with older adolescents being more affected by the pandemic [41, 42], perceiving negative changes in family relationships more strongly [41] and reporting poorer general (mental) health, both, before and during the pandemic [43, 44]. It is possible that physical and psychological development during puberty may favor more negative emotions in older adolescents. These age-differentiated findings can also be interpreted against the background of the concept of developmental tasks. These are normative age-typical tasks that result from biological development processes, social and societal demands and individual goals. Coping with these tasks not only has an impact on development in the further course of life, but also has implications for health behaviour (e.g. development of a health-promoting lifestyle) [45]. The development of social relationships with one’s own and the opposite sex, the adoption and development of a gender identity and role, the attainment of autonomy from parents and the development of intellectual and social skills are all relevant for childhood and adolescence [46]. Our results clearly show that older children and adolescents are less likely than younger children to perceive the positive developments in their family and circle of friends. This could be due to the fact that the need for autonomy increases with the age of the children, which was perceived as clearly limited as a result of the pandemic-related restrictions [22]. The situation is similar with the development of social relationships with friends, which are particularly important for older adolescents in connection with the desire for autonomy and were often limited to the digital space due to contact restrictions and the closure of leisure facilities [36]. In contrast to a typically desired reduction in parental control, a study from the Netherlands found an increase in parental control (in the form of corona-related rules) immediately after the lockdown [47].

With regard to gender-specific differences, these study results show that girls were more likely than boys to rate the effects of the corona measures as more negative in most areas of life, which is also confirmed in the literature [42, 48]. Girls report less support from family, peers and teachers and a less positive atmosphere at home [43]. One possible explanation for the gender difference could be that girls and boys have different subjective perceptions of their well-being [49]. In general, girls report poorer mental health more frequently than boys, even independently of crises, such as the coronavirus pandemic [50]. There are also indications that girls are more willing to express negative perceptions than boys [51]. For the first time, this study also looked at children and adolescents with gender diversity, who rated the effects of the measures on their lives significantly worse than children and adolescents with a binary gender identity, particularly in the area of mental health. Other studies have also shown that non-binary children and adolescents rate their health slightly worse, have lower life satisfaction and are more likely to report suffering from psychosomatic complaints [11]. This group was particularly affected by general and identity-specific stressors during the COVID-19 pandemic. In addition to a lack of family support, these include a lack of direct access to social networks and safe communities. While school closures are perceived as a loss of the social system on the one hand, distance learning is also associated with a decrease in discrimination and experiences of victimisation on the other hand [52, 53].

Furthermore, the available study results point to socioeconomic inequalities, with children and adolescents with a high level of family affluence more frequently reporting positive effects of the coronavirus pandemic in most areas of life. According to other studies, children from families with a high level of education were better able to cope with the current COVID-19 situation than was reported by children and adolescents with a low household income [31]. Pre-pandemic studies show that an already existing good standard of living benefits dealing with problem situations [54]. In addition, socially disadvantaged children were particularly burdened during the pandemic [12] and suffered more frequently from financial difficulties, which for example was associated with more mental health problems in the family [55, 56]. In addition, it was shown that low parental education also influences parenting practices and is less supportive in school tasks [57]. Socioeconomically disadvantaged children and adolescents have fewer resources at their disposal to cope with challenges in an already difficult life situation, which is more often characterised by poverty, limitations in leisure activities or family conflicts [58, 59]. Due to these social determinants, which are unequally distributed to their disadvantage, negative assessments of their own health are also more frequent and – as the present results confirm – health disadvantages in many areas of life.

###  

#### Strengths and limitations

The HBSC study is characterised by the fact that it uses a standardised procedure to regularly collect comprehensive health data from schoolchildren in a representative sample of 11-, 13- and 15-year-olds by means of self-reporting. For the first time in the 2022 HBSC survey wave, children and adolescents had the opportunity to use a third category for their gender information beyond the binary information ‘girl’ or ‘boy’. In order to be able to make reliable statements about this very small group of children and young people with gender diversity, additional analyses were carried out and evaluated in a gender-specific sub-sample with more balanced cell populations.

One of the limitations of this study is that no causal relationships could be investigated due to the cross-sectional design. The data from the survey from March to November 2022 was used in this evaluation. Due to the long survey period, possible effects in the dynamic course of the COVID-19 pandemic are possible. For example, the coronavirus wave that still prevailed in winter 2021/2022 and the associated restrictions may have had a greater impact on data collection in spring 2022 than at a later survey date (summer/autumn 2022). It is also possible that the (subjectively) perceived restrictions due to the pandemic were rated more negatively by children and adolescents in spring than in summer or fall when the pandemic situation eased somewhat. It was not possible to carry out a differentiated evaluation by federal state or district due to the very small subsamples in some federal states. The low response rate among schools (8.4 %) may also have been exacerbated by the pandemic.

The assessment of the subjective perception of the effects of the pandemic measures is both a strength and a weakness of the study. On the one hand, it enriches present research by a perspective that has received little attention so far. On the other hand, it is more prone to distorting influences, e.g. memory errors or the tendency to subsequently gloss over negative experiences (fading affect bias). Expanding the subjective perspective of children and adolescents by the parents’ perspective could enrich future research in this area.

#### Implications

The major finding of this study, namely that the subjective (retrospective) view of children and adolescents on the COVID-19 pandemic and its effects on their lives tends to be more positive than negative, makes an important contribution to the state of research on this topic. Studies with more objective indicators (e.g. standardised screening instruments for mental health) have so far tended to paint a less favourable picture in this regard. For future public health crises, a stronger and systematic consideration of the subjective perspective and its inclusion in research (participatory-based research) would therefore be desirable – also including particularly vulnerable groups, such as gender-diverse adolescents. This sometimes requires new online-based methods (e.g. ecological momentary assessment) that can be integrated into the everyday lives of young people, correspond to their mode of communication (e.g. audio messages or photos instead of questionnaires) and complement traditional surveys. It should also be noted that the effects analysed in the HBSC study should not be viewed irrespective of one another. For example, developmental tasks, such as detachment from parents and building social relationships are closely interwoven and coping with them is not independent of age, gender and socioeconomic status. Future studies should therefore pay more attention to the interaction of these factors in particular in order to enable the most concrete and target group-specific derivations possible for social situation-related public health.

Particularly relevant for the planning of health promotion measures in childhood and adolescence are the aspects in which the subjective assessments in this study and findings from studies with more objective indicators converge. For example, both perspectives are congruent in that the COVID-19 pandemic and the associated containment measures have particularly affected the mental health of children and adolescents, and that girls and older adolescents are more affected by this than boys and younger respondents. In addition, the data indicates that children and adolescents from families with a lower family affluence had more difficulties in almost all areas during the pandemic than children and adolescents from more privileged social backgrounds. However, it also shows that relationships with family and friends are an important protective factor and that crises can be better overcome with social support. This evidence, supplemented by the subjective perspective, should be the starting point for needs-orientated and target group-specific health promotion measures in these already known vulnerable groups.

## Figures and Tables

**Table 1:** Sample characteristics by gender, age group and socioeconomic status (*N* = 6,475)*. Source: HBSC Germany 2022

	*n*	%	*M (SD)*
**Gender**			
Girls	3,258	50.6	
Boys	3,074	47.7	
Gender diverse	112	1.7	
**Age group**			
11 years (5^th^ grade)	2,132	33.3	11.46 (0.39)
13 years (7^th^ grade)	2,160	33.7	13.46 (0.39)
15 years (9^th^ grade)	2,113	33.0	15.46 (0.38)
**Family affluence**			
Low	1,117	17.9	
Medium	4,191	67.4	
High	918	14.7	

*M* = Mean, *SD* = Standard deviation

*Absolute figures unweighted, percentages weighted, some of the data contain missing values in individual variables, so there may be deviations in the total number of cases.

**Annex Table 1:** Results of the chi-square test on the perception of a positive impact of the coronavirus pandemic on various areas of life, differentiated by age, gender and family affluence (FAS) (figures in %, *N* = 5,569 – 5,684). Source: HBSC Germany 2022

	Relationships with family	Relationships with friends	Physical activity	Family finances	Overall health	Eating behaviour	Future expectations	School performance	Life in general	Mental health
**Age group**										
*N*	5,789	5,750	5,780	5,692	5,794	5,756	5,726	5,786	5,813	5,757
11-year-olds (*n* = 1,806 – 1,866)	67.6	58.3	55.5	57.1	55.2	56.2	49.2	44.9	45.1	44.5
13-year-olds (*n* = 1,952 – 1,996)	54.8	51.6	44.4	43.5	41.2	38.1	37.7	33.4	31.6	30.7
15-year-olds (*n* = 1,934 – 1,952)	42.0	45.0	36.4	28.4	27.3	28.6	27.7	28.3	25.4	22.2
*p*	< 0.001	< 0.001	< 0.001	< 0.001	< 0.001	< 0.001	< 0.001	< 0.001	< 0.001	< 0.001
V	0.158	0.098	0.111	0.176	0.164	0.165	0.127	0.125	0.125	0.154
**Gender**										
*N*	5,793	5,754	5,785	5,696	5,799	5,757	5,727	5,791	5,819	5,760
Girls (*n* = 2,842 – 2,901)	53.1	51.5	42.6	41.6	38.0	39.0	36.5	35.2	31.0	28.4
Boys (*n =* 2,753 – 2,816)	57.5	51.9	49.0	44.6	45.0	43.3	40.4	36.1	37.6	37.0
Gender diverse (*n* = 101 – 102)	21.8	40.6	17.6	21.8	20.6	11.8	10.9	21.6	11.8	9.9
*p*	< 0.001	0.106	< 0.001	< 0.001	< 0.001	< 0.001	< 0.001	< 0.001	< 0.001	< 0.001
V	0.080	0.026	0.075	0.046	0.089	0.098	0.090	0.051	0.078	0.155
**Family affluence**										
*N*	5,656	5,629	5,655	5,569	5,664	5,623	5,601	5,654	5,684	5,625
High (*n* = 829 – 849)	62.0	55.6	58.5	51.0	46.1	44.1	45.2	40.1	41.0	37.5
Medium (*n* = 3,752 – 3,823)	53.1	51.3	43.5	41.6	40.0	39.9	36.8	33.9	32.9	30.9
Low (*n* = 988 – 1,013)	53.7	49.4	39.7	39.3	40.0	40.2	36.7	36.9	31.0	32.3
*p*	< 0.001	< 0.001	< 0.001	< 0.001	< 0.001	0.242	< 0.001	0.010	< 0.001	< 0.001
V	0.059	0.042	0.086	0.062	0.046	0.022	0.054	0.034	0.050	0.040

**Annex Table 2:** Results of the binary-logistic regression to predict a subjectively negative influence of the corona measures on various areas of life (*n* = 3,258 girls, *n* = 3,074 boys, without gender-diverse category). Source: HBSC Germany 2022

	Relationships with family (*n* = 5,518)	Relationships with friends (*n* = 5,501)	Physical activity (*n* = 5,504)	Family finances (*n* = 5,429)	Overall health (*n* = 5,516)	Eating behaviour (*n* = 5,477)	Future expectations (*n* = 5,449)	School performance (*n* = 5,602)	Life in general (*n* = 5,533)	Mental health (*n* = 5,484)
	OR (95 %-CI)	OR (95 %-CI)	OR (95 %-CI)	OR (95 %-CI)	OR (95 %-CI)	OR (95 %-CI)	OR (95 %-CI)	OR (95 %-CI)	OR (95 %-CI)	OR (95 %-CI)
**Age group**										
11-year-olds (Ref.)										
13-year-olds	1.12 (0.93 – 1.36)	0.93 (0.79 – 1.10)	1.37 (1.18 – 1.60)^***^	1.42 (1.16 – 1.74)^***^	1.13 (0.95 – 1.36)	1.84 (1.53 – 2.21)^***^	1.17 (0.97 – 1.41)	1.91 (1.63 – 2.24)^***^	1.04 (0.89 – 1.23)	2.05 (1.76 – 2.39)^***^
15-year-olds	1.15 (0.94 – 1.39)	0.85 (0.72 – 1.01)	1.53 (1.31 – 1.78)^***^	1.09 (0.88 – 1.35)	1.44 (1.21 – 1.72)^***^	1.96 (1.63 – 2.36)^***^	1.33 (1.11 – 1.60)^**^	2.42 (2.07 – 2.82)^***^	1.34 (1.14 – 1.56)^***^	2.68 (2.31 – 3.12)^***^
**Gender**										
Boys (Ref.)										
Girls	1.51 (1.29 – 1.76)^***^	1.12 (0.98 – 1.29)	1.09 (0.97 – 1.24)	1.01 (0.86 – 1.19)	1.54 (1.33 – 1.78)^***^	1.73 (1.50 – 2.00)^***^	1.24 (1.07 – 1.43)^**^	1.11 (0.98 – 1.25)	1.19 (1.05 – 1.36)^**^	2.43 (2.16 – 2.74)^***^
**Family affluence**										
High (Ref.)										
Medium	1.38 (1.08 – 1.77)^*^	1.30 (1.05 – 1.61)^*^	1.72 (1.42 – 2.10)^***^	1.43 (1.09 – 1.88)^**^	1.10 (0.89 – 1.36)	1.08 (0.88 – 1.33)	1.18 (0.95 – 1.47)	1.23 (1.03 – 1.47)^*^	1.31 (1.08 – 1.60)^**^	1.25 (1.05 – 1.49)^*^
Low	1.89 (1.42 – 2.51)^***^	1.72 (1.34 – 2.20)^***^	2.11 (1.68 – 2.65)^***^	2.07 (1.52 – 2.81)^***^	1.50 (1.17 – 1.92)^**^	1.13 (0.88 – 1.44)	1.58 (1.22 – 2.05)^***^	1.14 (0.92 – 1.42)	1.49 (1.18 – 1.88)^***^	1.26 (1.02 – 1.56)^*^
Nagelkerke R^2^	0.02	0.01	0.02	0.01	0.02	0.04	0.01	0.04	0.01	0.10

OR = Odds Ratio, CI = Confidence interval, Ref. = Reference group,

^*^p < 0.05,

^**^p < 0.01,

^***^p < 0.001

**Annex Table 3:** Results of the binary-logistic regression to predict a subjectively positive influence of the coronavirus measures on various areas of life (*n* = 3,258 girls, *n* = 3,074 boys, without gender-diverse category). Source: HBSC Germany 2022

	Relationships with family (*n* = 5,518)	Relationships with friends (*n* = 5,501)	Physical activity (*n* = 5,504)	Family finances (*n* = 5,429)	Overall health (*n* = 5,516)	Eating behaviour (*n* = 5,477)	Future expectations (*n* = 5,449)	School performance (*n* = 5,505)	Life in general (*n* = 5,533)	Mental health (*n* = 5,484)
	OR (95 %-CI)	OR (95 %-CI)	OR (95 %-CI)	OR (95 %-CI)	OR (95 %-CI)	OR (95 %-CI)	OR (95 %-CI)	OR (95 %-CI)	OR (95 %-CI)	OR (95 %-CI)
**Age group**										
11-year-olds (Ref.)										
13-year-olds	0.58 (0.51 – 0.66)^***^	0.75 (0.66 – 0.86)^***^	0.63 (0.56 – 0.72)^***^	0.57 (0.50 – 0.65)^***^	0.56 (0.50 – 0.64)^***^	0.48 (0.42 – 0.55)^***^	0.61 (0.54 – 0.70)^***^	0.61 (0.53 – 0.70)^***^	0.56 (0.49 – 0.64)^***^	0.55 (0.48 – 0.63)^***^
15-year-olds	0.35 (0.30 – 0.40)^***^	0.58 (0.51 – 0.66)^***^	0.46 (0.40 – 0.52)^***^	0.29 (0.26 – 0.34)^***^	0.30 (0.26 – 0.34)^***^	0.31 (0.27 – 0.36)^***^	0.40 (0.35 – 0.46)^***^	0.49 (0.43 – 0.56)^***^	0.43 (0.37 – 0.49)^***^	0.35 (0.30 – 0.41)^***^
**Gender**										
Boys (Ref.)										
Girls	0.84 (0.75 – 0.93)^**^	0.99 (0.89 – 1.11 )	0.79 (0.71 – 0.88)^***^	0.91 (0.81 – 1.01)	0.74 (0.66 – 0.83)^***^	0.84 (0.75 – 0.94)^**^	0.88 (0.79 – 0.98)^*^	0.97 (0.87 – 1.09)	0.75 (0.67 – 0.84)^***^	0.68 (0.61 – 0.76)^***^
**Family affluence**										
High (Ref.)										
Medium	0.70 (0.60 – 0.82)^***^	0.84 (0.72 – 0.97)^*^	0.54 (0.47 – 0.63)^***^	0.67 (0.58 – 0.79)^***^	0.79 (0.67 – 0.92)^**^	0.85 (0.73 – 0.99)^*^	0.70 (0.60 – 0.82)^***^	0.76 (0.65 – 0.89)^***^	0.71 (0.61 – 0.83)^***^	0.76 (0.65 – 0.89)^***^
Low	0.71 (0.59 – 0.86)^***^	0.76 (0.63 – 0.92)^**^	0.46 (0.38 – 0.55)^***^	0.61 (0.50 – 0.74)^***^	0.78 (0.64 – 0.94)^*^	0.84 (0.69 – 1.02)	0.70 (0.57 – 0.84)^***^	0.86 (0.71 – 1.04)	0.65 (0.54 – 0.79)^***^	0.82 (0.67 – 0.99)^*^
Nagelkerke R^2^	0.06	0.02	0.05	0.08	0.08	0.07	0.05	0.03	0.05	0.07

OR = Odds Ratio, CI = Confidence interval, Ref. = Reference group,

^*^p < 0.05,

^**^p < 0.01,

^***^p < 0.001

**Annex Table 4:** Results of the binary-logistic regression to predict a subjectively negative influence of the corona measures on various areas of life in the subsample analysis (*n* = 300 girls, *n* = 300 boys, *n* = 112 gender diverse). Source: HBSC Germany 2022

	Relationships with family (*n* = 630)	Relationships with friends (*n* = 631)	Physical activity (*n* = 632)	Family finances (*n* = 626)	Overall health (*n* = 632)	Eating behaviour (*n* = 630)	Future expectations (*n* = 624)	School performance (*n* = 631)	Life in general (*n* = 632)	Mental health (*n* = 628)
	OR (95 %-CI)	OR (95 %-CI)	OR (95 %-CI)	OR (95 %-CI)	OR (95 %-CI)	OR (95 %-CI)	OR (95 %-CI)	OR (95 %-CI)	OR (95 %-CI)	OR (95 %-CI)
**Age group**										
11-year-olds (Ref.)										
13-year-olds	1.01 (0.54 – 1.92)	0.82 (0.47 – 1.44)	1.23 (0.78 – 1.95)	0.84 (0.45 – 1.59)	1.21 (0.66 – 2.22)	1.75 (0.99 – 3.08)	1.14 (0.65 – 2.02)	1.62 (0.99 – 2.67)	1.17 (0.71 – 1.93)	1.52 (0.95 – 2.44)
15-year-olds	1.45 (0.81 – 2.59)	1.17 (0.70 – 1.95)	1.25 (0.80 – 1.94)	0.88 (0.49 – 1.61)	1.43 (0.81 – 2.52)	2.38 (1.40 – 4.03)^**^	1.54 (0.91 – 2.61)	2.84 (1.78 – 4.50)^***^	2.09 (1.32 – 3.30)^**^	2.57 (1.65 – 4.00)^***^
**Gender**										
Boys (Ref.)										
Girls	1.26 (0.75 – 2.14)	1.29 (0.81 – 2.04)	0.93 (0.63 – 1.37 )	1.10 (0.64 – 1.90)	1.26 (0.76 – 2.10)	1.45 (0.93 – 2.25)	0.96 (0.60 – 1.54)	1.31 (0.88 – 1.93)	0.76 (0.51 – 1.14)	2.34 (1.59 – 3.44)^***^
Gender diverse	2.92 (1.59 – 5.37)^***^	1.49 (0.81 – 2.76)	1.96 (1.19 – 3.22)^**^	1.60 (0.80 – 3.19)	4.55 (2.57 – 8.09)^***^	2.40 (1.39 – 4.14)^**^	2.75 (1.59 – 4.73)^***^	2.24 (1.35 – 3.72)^**^	1.87 (1.13 – 3.09)^*^	5.26 (3.12 – 8.86)^***^
**Family affluence**										
High (Ref.)										
Medium	1.29 (0.65 – 2.59)	0.97 (0.54 – 1.73)	1.11 (0.67 – 1.83)	0.67 (0.35 – 1.28)	0.95 (0.51 – 1.75)	1.08 (0.62 – 1.88)	0.90 (0.51 – 1.59)	0.82 (0.51 – 1.31)	1.11 (0.67 – 1.85)	1.00 (0.62 – 1.63)
Low	2.07 (0.93 – 4.60)	1.34 (0.66 – 2.72)	1.71 (0.93 – 3.13)	1.32 (0.62 – 2.82)	1.29 (0.62 – 2.70)	1.11 (0.55 – 2.22)	1.31 (0.66 – 2.60)	0.77 (0.41 – 1.42)	1.43 (0.76 – 2.68)	1.23 (0.67 – 1.27)
Nagelkerke R^2^	0.06	0.02	0.04	0.02	0.10	0.06	0.07	0.09	0.07	0.16

OR = Odds Ratio, CI = Confidence interval, Ref. = Reference group,

^*^p < 0.05,

^**^p < 0.01,

^***^p < 0.001

**Annex Table 5:** Results of the binary-logistic regression to predict a subjectively positive influence of the corona measures on various areas of life in the subsample analysis (*n* = 300 girls, *n* = 300 boys, *n* = 112 gender diverse). Source: HBSC Germany 2022

	Relationships with family (*n* = 630)	Relationships with friends (*n* = 631)	Physical activity (*n* = 632)	Family finances (*n* = 626)	Overall health (*n* = 632)	Eating behaviour (*n* = 630)	Future expectations (*n* = 624)	School performance (*n* = 631)	Life in general (*n* = 632)	Mental health (*n* = 628)
	OR (95 %-CI)	OR (95 %-CI)	OR (95 %-CI)	OR (95 %-CI)	OR (95 %-CI)	OR (95 %-CI)	OR (95 %-CI)	OR (95 %-CI)	OR (95 %-CI)	OR (95 %-CI)
**Age group**										
11-year-olds (Ref.)										
13-year-olds	0.43 (0.28 – 0.66)^***^	0.63 (0.42 – 0.95)^*^	0.70 (0.46 – 1.05)	0.60 (0.40 – 0.91)^*^	0.61 (0.40 – 0.92)^*^	0.52 (0.34 – 0.79)^**^	0.58 (0.38 – 0.88)^*^	0.58 (0.38 – 0.89)^*^	0.48 (0.31 – 0.74)^***^	0.51 (0.33 – 0.80)^**^
15-year-olds	0.25 (0.17 – 0.38)^***^	0.45 (0.31 – 0.68)^***^	0.39 (0.26 – 0.59)^***^	0.26 (0.17 – 0.40)^***^	0.31 (0.20 – 0.47)^***^	0.35 (0.23 – 0.53)^***^	0.27 (0.18 – 0.43)^***^	0.42 (0.28 – 0.64)^***^	0.35 (0.23 – 0.54)^***^	0.38 (0.24 – 0.58)^***^
**Gender**										
Boys (Ref.)										
Girls	0.90 (0.63 – 1.29)	1.03 (0.73 – 1.46)	1.07 (0.76 – 1.52)	0.95 (0.66 – 1.36)	0.92 (0.64 – 1.31)	0.87 (0.61 – 1.24)	0.90 (0.62 – 1.30)	0.82 (0.57 – 1.18)	0.89 (0.62 – 1.29)	0.60 (0.41 – 0.88)^**^
Gender diverse	0.35 (0.20 – 0.61)^***^	0.82 (0.50 – 1.34)	0.46 (0.27 – 0.81)^**^	0.57 (0.32 – 0.99)^*^	0.43 (0.24 – 0.77)^**^	0.31 (0.17 – 0.58)^***^	0.45 (0.24 – 0.83)^*^	0.60 (0.34 – 1.06)	0.40 (0.21 – 0.77)^**^	0.30 (0.15 – 0.58)^***^
**Family affluence**										
High(Ref.)										
Medium	1.18 (0.74 – 1.86)	1.10 (0.71 – 1.70)	0.91 (0.58 – 1.43)	0.69 (0.44 – 1.10)	0.89 (0.56 – 1.41)	0.89 (0.56 – 1.41)	0.67 (0.42 – 1.08)	0.79 (0.50 – 1.26)	0.80 (0.50 – 1.28)	0.95 (0.58 – 1.53)
Low	1.11 (0.62 – 2.00)	0.97 (0.55 – 1.69)	0.62 (0.34 – 1.12)	0.55 (0.30 – 1.01)	0.86 (0.48 – 1.55)	0.90 (0.49 – 1.63)	0.71 (0.38 – 1.31)	0.71 (0.39 – 1.30)	0.53 (0.28 – 0.99)^*^	0.59 (0.31 – 1.13)
Nagelkerke R^2^	0.15	0.04	0.09	0.12	0.11	0.11	0.12	0.06	0.10	0.11

OR = Odds Ratio, CI = Confidence interval, Ref. = Reference group,

^*^p < 0.05,

^**^p < 0.01,

^***^p < 0.001
